# Complete chloroplast genome of *Lamium takesimese* Nakai (Lamiaceae): an endemic species in South Korea

**DOI:** 10.1080/23802359.2019.1667899

**Published:** 2019-09-23

**Authors:** Kyu Tae Park, Jaehang Shin, Seonjoo Park

**Affiliations:** a Department of Life Sciences, Yeungnam University, Gyeongsan, Gyeongbuk, South Korea;; b Department of Biomedical Informatics, University of Utah School of Medicine, Salt Lake City, UT, USA

**Keywords:** *Lamium takesimense*, Lamiaceae, complete chloroplast genome, endemic species,, Ulleung island

## Abstract

The complete chloroplast genome of *Lamium takesimense* (Lamiaceae), an endemic species in South Korea, are presented in this article. The genome size is 150,626 bp in length, with 38.6% of GC content. It consists a large single-copy (LSC) region (82,527bp) and a small single-copy region (SSC) (17,185bp) which were separated by two inverted repeat (IRs) regions (25,457bp). The complete chloroplast genome contains 111 unique genes, including 77 protein-coding genes, 4 rRNA genes, and 30 tRNA genes.

*Lamium* L. is the type genus of family Lamiaceae and contains approximately 40–50 species. The genus is native to the temperate and subtropical regions of Europe, Asia, and Northern Africa (Bendiksby et al. 2011). This genus is useful medicine resources for antispasmodic, astringent, anti-proliferative, anti-inflammatory and antiviral (Yalcin and Kaya 2006). One of the species of *Lamium* L, *Lamium takesimense* Nakai (Lamiaceae), is known as an endemic species in Korea and its habitat is restricted on Ulleung Island in East Sea. This work aims to determine the complete chloroplast genome sequence of *L.* takesimense and contribute to provide genetic information of endemic species.

*Lamium takesimense* was collected from Ulleng Island (N37°29′20″, E130°53′56″). And the voucher (YNUH16U128) was stored at Yeungnam University Herbarium (YNUH). The total DNA was isolated using the DNeasy plant Mini Kit (Quiagen, Carlsbad, CA). The complete chloroplast genome of *L. takesimense* was sequenced by HiSeq2000 sequencer of Illumina (San Diego, CA), *de novo* assembled with SOAPdenovo2 (Luo et al. 2012). The annotation was conducted using DOGMA (Wyman et al. 2004) and CpGAVAS (Liu et al. 2012). Also, tRNA were confirmed with tRNAscan-SE (Lowe and Eddy 1997).

The complete chloroplast genome sequence of *L. takesimense* was 150,626 bp and deposited in GenBank (MN240520). It consists of one LSC (82,527bp), one SSC (17,185bp), and two IRs (25,457bp). The overall GC contents of cp genome were 38.6% and in the LSC, SSC, and IRs were 36.8%, 32.9% and 43.4%, respectively.

The chloroplast genome contains 111 unique genes, including 77 protein coding genes, 4 rRNA genes, and 30 tRNA genes. Among these genes, sixteen genes (*atpF, clpP, ndhA, ndhB, rpoC1, rps12, rps16, rpl2, ycf3, trnA-*UGC*, trnG-*UUC*, trnI-*GAU*, trnK-*UUU*, trnL-*UAA*, trnV-*UAC) contained one or two introns. And nineteen of those genes were duplicated in IR regions (*ndhB, rpl2, rpl23, rps12, rps7, rps19, ycf2, ycf15, rrn4.5 rrn5, rrn16, rrn23, trnA-*UGC*, trnI-*CAU*, trnI-*GAU*, trnL-*CAA, *trnN-*GUU, *trnR-*ACG, *trnV*-GAC). The Maximum Likelihood phylogenetic tree was generated using RAxML (Stamatakis 2014) based on the complete chloroplast genome of *L. takesimense* and fourteen other species from Lamiaceae (13 Lamioideae, and 1 Scutellaroideae as an outgroup). Most nodes in a phylogenetic tree were supported strongly. And the phylogenetic tree showed that *L. takesimense* was closely related to *Lamium album* (Figure 1). The complete chloroplast sequence of *L. takesimense* will provide a useful information for phylogenetic and evolutionary studies in *Lamium* and its related species.

**Figure 1.**
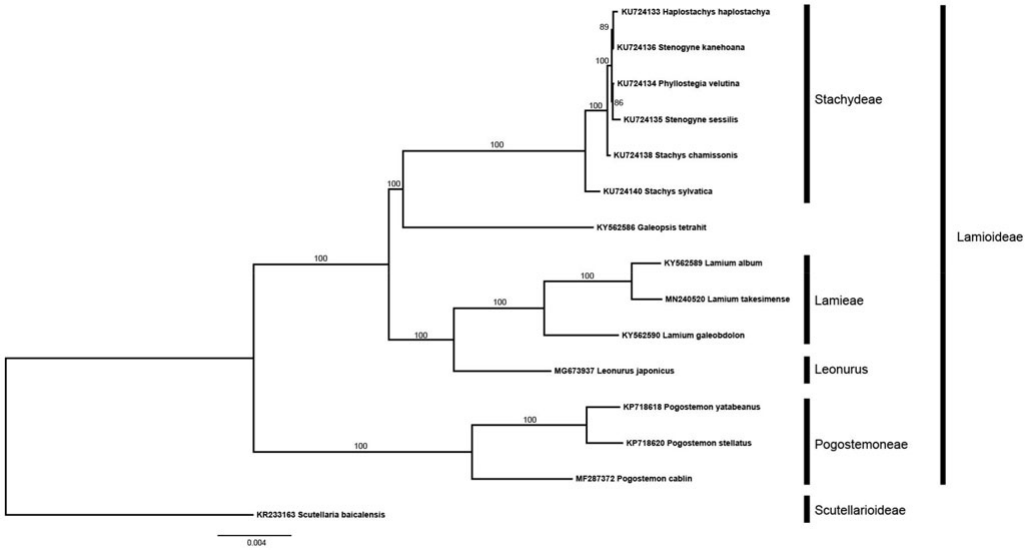
Maximum Likelihood phylogenetic tree generated by RAxML based on complete chloroplast genome sequence of 15 species from the family Lamiaceae. The bootstrap value based on 1000 replicates is shown on branches.
